# The identification and prediction of frailty based on Bayesian network analysis in a community-dwelling older population

**DOI:** 10.1186/s12877-022-03520-7

**Published:** 2022-11-11

**Authors:** Yin Yuan, Siyang Lin, Xiaoming Huang, Na Li, Jiaxin Zheng, Feng Huang, Pengli Zhu

**Affiliations:** 1 Department of Geriatric Medicine, Fujian Provincial Hospital, Fuzhou, China; 2 Shengli Clinical Medical College, Fujian Medical University, Fuzhou, China; 3 Fujian Provincial Institute of Clinical Geriatrics, Fuzhou, China; 4 Fujian Provincial Center of Geriatrics, Fuzhou, China; 5 Fujian Provincial Key Laboratory of Geriatrics, Fuzhou, China

**Keywords:** Bayesian analysis, Frail older adults, Risk factors

## Abstract

**Background:**

We have witnessed frailty, which characterized by a decline in physiological reserves, become a major public health issue in older adults. Understanding the influential factors associated with frailty may help prevent or if possible reverse frailty. The present study aimed to investigate factors associated with frailty status and frailty transition in a community-dwelling older population.

**Methods:**

A prospective cohort study on community-dwelling subjects aged ≥ 60 years was conducted, which was registered beforehand (ChiCTR 2,000,032,949). Participants who had completed two visits during 2020–2021 were included. Frailty status was evaluated using the Fried frailty phenotype. The least absolute shrinkage and selection operator (LASSO) regression was applied for variable selection. Bayesian network analysis with the max-min hill-climbing (MMHC) algorithm was used to identify factors related to frailty status and frailty transition.

**Results:**

Of 1,981 subjects at baseline, 1,040 (52.5%) and 165 (8.33%) were classified as prefrailty and frailty. After one year, improved, stable, and worsening frailty status was observed in 460 (35.6%), 526 (40.7%), and 306 (23.7%) subjects, respectively. Based on the variables screened by LASSO regression, the Bayesian network structure suggested that age, nutritional status, instrumental activities of daily living (IADL), balance capacity, and social support were directly related to frailty status. The probability of developing frailty is 14.4% in an individual aged ≥ 71 years, which increases to 20.2% and 53.2% if the individual has balance impairment alone, or combined with IADL disability and malnutrition. At a longitudinal level, ADL/IADL decline was a direct predictor of worsening in frailty state, which further increased the risk of hospitalization. Low high-density lipoprotein cholesterol (HDL-C) and diastolic blood pressure (DBP) levels were related to malnutrition, and further had impacts on ADL/IADL decline, and ultimately led to the worsening of the frailty state. Knowing the status of any one or more of these factors can be used to infer the risk of frailty based on conditional probabilities.

**Conclusion:**

Older age, malnutrition, IADL disability, and balance impairment are important factors for identifying frailty. Malnutrition and ADL/IADL decline further predict worsening of the frailty state.

**Supplementary Information:**

The online version contains supplementary material available at 10.1186/s12877-022-03520-7.

## Background

Frailty is an age-related geriatric syndrome characterized by increased vulnerability to stress and a decline in physiological reserves, involving the dysfunction of neuromuscular, metabolic and immune systems [1]. The cumulative decline erodes the physical homeostatic reserve, until minor stressor events trigger changes in health status, and lead to a series of clinical negative events such as functional limitations, institutionalization, and death [2].

A better grasp of frailty associated factors help prevent or if possible reverse frailty. Evidence is accumulating that various factors including disability, cognitive impairment, living alone, functional dependence, malnutrition, and prior history of falls and hospitalization increased frailty risks [3–5]. The identification of modifiable contributing factors can assist in designing individualized interventions that focused on improving frailty status, and preventing the development of adverse outcomes in early stages [6]. However, most of the factors were reported from a cross-sectional level. Frailty is virtually considered as a dynamic state that can worsen or improve over time. A dramatic deterioration of frailty may occur in a very short period of time for high-risk elder individuals without proper intervention [4], therefor assessing predictive factors of frailty progression is critical as well. Furthermore, taking into account of time-dependent association could provide more information when analyzing factors that relate to frailty transition, but there is still a lack of longitudinal studies investigating in this field.

Frailty is multifactorial, and there are also intricate relationships among its influencing factors [7]. Traditional statistical models only focus on displaying correlations between dependent and various independent variables without reflecting the overall linkage effect [8]. Compared with logistic regression, Bayesian network intuitively describes the correlations between variables by constructing directed acyclic graphs, and also allow us to obtain the correlation strength by conditional probability calculation [9]. Bayesian network can also make inferences of unknown node according to the state of a known node, so as to be applied in risk assessment [10]. Da Cunha Leme found that multiple health factors were associated with frailty, falls, and hospitalization events in a complex network [7], suggesting the Bayesian network that can help analyze the complex phenomena and dynamic changes of frailty.

The present study was designed to investigate influential factors of frailty status and the predictors of frailty transition from a cross-sectional and longitudinal level based on discrete Bayesian network topology. The results were expected to guide the clinical decision making for frailty risk assessment and strategies establishment in a community-dwelling older population.

## Methods

### Study design and participants

The study of “Fujian prospective aging cohort”, which was registered and initially took place in 2020, was conducted to investigate the health status based on comprehensive geriatric assessment and cardiovascular events in non-hospitalized older population (ChiCTR 2,000,032,949). The study recruited residents aged 60 years and above from community and nursing homes in Fujian Province, who were followed-up every 1 to 2 years. Written informed consent was obtained from all participants prior to their enrollment. Subjects were excluded from this study if they had (a) less than six months of life expectancy due to critical illness or advanced malignancy, (b) long-term bedridden or been complete disability, and (c) severe visual, hearing or language communication disorders to cooperate with questionnaire investigation and physical examination.

This current study included subjects who had completed baseline and one-year follow-up surveys. We firstly enrolled 2,044 subjects from Wenquan Community, Fuzhou City in May 2020, and 1,981 (aged 60–98 years, 1189 females and 792 males) was included in the baseline analysis. The second visit was conducted in July 2021, during which 1,292 subjects completed. Supplementary Fig. 1 displays the flowchart of the study. Supplementary Table 1 presents the comparison of the characteristics of subjects who did or did not complete two visits. Participants were comparable, except those completed the visits had a higher proportion of living alone (*P* = 0.024). Supplementary Table 2 shows the characteristics of the subjects at baseline and after one year. This study was evaluated and approved by the Ethics Committee of Fujian Provincial Hospital (No. K2020-05-008) and was conducted according to the principles of the Declaration of Helsinki.

## Measurements

### Frailty phenotype

Frailty status was defined according to the Fried phenotype [11]. The components include unintentional weight loss (at least 5% of previous year’s body weight), self-reported exhaustion, slow walking speed (the slowest 20% based on 15-feet of walking time, adjusting for gender and standing height), weakness (the lowest 20% of grip strength stratified by gender and BMI quartiles) and low physical activity level (expenditure of physical activity per week < 383 kcal for men, < 270 kcal for women). Subjects who did not score in any of the components were classified as “non-frail”, while those who scored in one or two components were classified as “pre-frail”, and those who scored in three or more as “frail”. After one year of follow-up, the frailty status was reevaluated. The change of the score of frailty components was calculated by baseline score minus follow-up score. Participants who received negative scores were considered as “worsening of the frailty state”, while those who received positive scores or zero were considered as “improved or stable frailty state”.

### Covariates

The following factors were considered to be covariates, which were obtained from comprehensive questionnaires: (a) demographic characteristics including age, gender, education, marital status, living condition, and monthly income; (b) lifestyle factors such as tobacco and alcohol use, and exercise habits; (c) clinical indicators including physician-diagnosed medical conditions, number of regular medications, hospital admissions and history of falls in the past one year; (d) physical function assessment on basic/instrumental activities of daily living (ADL/IADL) evaluated by the Katz scale [12] and Lawton instrumental activities of daily living scale [13]; balance capacity evaluated with the timed up and go test [14]; (e) sensory assessments including vision, hearing, and continence, answering “normal, mild impaired, impaired” on a three-point scale; (f) a psychophysiological assessment in which depression, anxiety, and insomnia were assessed by the geriatric depression scale-4 (GDS-4) [15], generalized anxiety disorder scale-7 (GAD-7) [16], and Athens insomnia scale (AIS) [17], respectively; chronic pain assessed with the numerical rating scale (NRS) [18]; (g) cognitive function evaluated using the minicog scale [19]; (h) dietary diversity score focusing on nine types of food including grains, vegetables, fruits, meat, seafood, eggs, dairy products, beans, and tea. The dietary diversity score was calculated based on the sum of the frequency of each type of food. Food frequency was defined as “not eaten = 0, yearly consumption = 1, monthly consumption = 2, weekly consumption = 3, daily consumption = 4”. (i) Social support was evaluated by the social support rating scale (SSRS); (j) the social environment and home safety were evaluated; (k) a physical examination was performed to assess height, weight, waist circumference, heart rate, office blood pressure, grip strength, and gait speed; (l): laboratory indicators including hemoglobin, albumin, creatinine, blood lipids, fasting blood glucose, and uric acid.

### Statistical analysis

Continuous variables are presented as mean ± standard deviation (SD) or medians ± 25th -75th percentiles. Categorical variables are reported as proportions. The characteristics of subjects according to different frailty status were compared using Student’s t test or analysis of variance (ANOVA) for variables with a normal distribution, the Mann-Whitney U or Kruskal-Wallis test for variables with a skewed distribution, and the chi-squared test for categorical parameters.

The LASSO (least absolute shrinkage and selection operator) algorithm for a logistic regression model using 10-fold cross-validation was applied for variable selection. Baseline frailty status or frailty transition (worsening or stable/improved) were taken as the dependent variables, and all variables were considered as potential confounders (Supplementary Table 3). LASSO penalizes parameter estimates generated using L1 penalization, and can find the optimal shrinkage parameter λ [20]. The models with minimum λ and one SD λ were compared in terms of discriminability and calibration. For discriminability, the C-statistic was compared with Delong tests. Calibration plots and Hosmer-Lemeshow goodness of fit were assessed to evaluate calibration. The model with the optimal λ was selected.

A Bayesian network is a directed acyclic graph consisting of nodes representing the variables X = {Xi, …, Xn} and directed edges representing the correlations between the variables [21]. On the edge of Xi→Xj, variable Xj is called the parent and Xi is the child of Xj. Each node has a conditional probability distribution table that measures the probability of its parent nodes. The Bayesian network represents the joint distribution of a set of random variables, and its joint probability P (X1,⋯,Xn) can be expressed as a Bayesian network B = (G,θ), in which G is the structure that demonstrates the random variables and the relations between them and θ is the conditional probability of each variable according to the structure G [8]. Bayesian network learning includes structure learning and parameter learning. In the present study, the max-min hill-climbing (MMHC) algorithm was used for structure learning. After creating the Bayesian network topology, maximum likelihood estimation (MLE) was applied to estimate the conditional probability of each node. Furthermore, 10-fold cross-validation analysis was performed. The dataset was randomly divided into ten equal subsamples, and in any ten-repeat cross-validation, nine subsamples were used to train the model, and the remaining one subsample was used to validate the model. The graph and inference model of the Bayesian network were drawn by Netica (Norsys Software Corp., Vancouver, BC, Canada).

A two-sided p value < 0.05 was considered statistically significant. Cross-validated LASSO regression were analyzed with R version 4.1.3 using the “glmnet” package, while Bayesian network analysis was performed using the “bnlearn” and “Rgraphviz” packages. All other analyses were performed with StataMP 15.1 (StataCorp, College Station, TX).

## Results

A total of 1,981 subjects (mean age = 72.5 years, 60.0% female) were included in baseline analysis. After one year, 1,292 participants had completed two visits. Of 1,981 subjects at baseline, 1,040 (52.5%) and 165 (8.3%) subjects were classified as prefrailty and frailty according to the Fried phenotype.

The baseline characteristics of participants according to the Fried phenotype are shown in Table 1. Prefrail and frail subjects were older, had a lower education level, were more likely to be living alone and widowed, and had higher risks of falling and balance impairment (all *P* < 0.001). They also had a lower dietary diversity score, ADL/IADL score, social support rating score, DBP and TC levels, (all *P* < 0.001), whereas they had higher proportions of being female, malnutrition, continence, constipation, hypertension, comorbidity, polypharmacy, cognitive impairment, anxiety, depression, insomnia, chronic pain, and higher levels of SBP, waist circumference, and creatinine (all *P* < 0.01).

**Table 1:** Baseline characteristics of subjects according to frail status

Variables		Robust (N = 776)	Prefrailty (N = 1,040)	Frailty (N = 165)	*P* value
**General Characteristics**					
Age (year, ‾x ± s)		70.3 ± 5.6	72.9 ± 7.2	80.2 ± 7.3	**< 0.001**
Female (N, %)		424 (54.6%)	650 (62.5%)	115 (69.7%)	**< 0.001**
Living alone (N, %)		40 (5.2%)	69 (6.6%)	18 (10.9%)	**< 0.001**
Widowed, divorced, unmarried (N, %)		128 (16.5%)	227 (21.8%)	68 (41.2%)	**< 0.001**
Education (primary school or below, N, %)		134 (17.3%)	211 (20.3%)	54 (32.7%)	**< 0.001**
**Nutrition & physical function**					
Dietary diversity score		29 (26, 32)	28 (24, 31)	26 (23, 30)	**< 0.001**
Malnutrition risk (N, %)		95 (12.2%)	259 (24.9%)	82 (49.7%)	**< 0.001**
ADL score (median, IQR)		6 (6, 6)	6 (6, 6)	6 (6, 6)	**< 0.001**
IADL score (median, IQR)		8 (8, 8)	8 (8, 8)	8 (4, 8)	**< 0.001**
Continence (n, %)		46 (5.9%)	114 (11.0%)	35 (21.2%)	**< 0.001**
Constipation (n, %)		14 (1.8%)	36 (3.5%)	10 (6.1%)	**0.007**
Unexplainable fall ≥ two times (N, %)		42 (5.4%)	111 (10.7%)	49 (29.7%)	**< 0.001**
**Medical condition & social support**					
Hypertension (N, %)		454 (58.5%)	676 (65.0%)	112 (67.9%)	**0.006**
Diabetes (N, %)		230 (29.6%)	344 (33.1%)	63 (38.2%)	0.067
Comorbidity (N, %)		2 (1, 3)	2 (1, 3)	2 (1, 4)	**< 0.001**
Polypharmacy (N, %)		186 (24.0%)	315 (30.3%)	78 (47.3%)	**< 0.001**
Cognitive impairment (N, %)		81 (10.4%)	194 (18.7%)	61 (37.0%)	**< 0.001**
Anxiety (N, %)		117 (15.1%)	266 (25.6%)	64 (38.8%)	**< 0.001**
Depression (N, %)		45 (5.8%)	97 (9.3%)	32 (19.4%)	**< 0.001**
Insomnia (N, %)		261 (33.6%)	422 (40.6%)	80 (48.5%)	**< 0.001**
Chronic pain (N, %)		293 (37.8%)	513 (49.4%)	113 (68.5%)	**< 0.001**
SSRS (median, IQR)		28 (24, 32)	27 (22, 31)	23 (19, 27)	**< 0.001**
**Physical Exam & Laboratory data**					
SBP (mmHg)		137 (126, 148)	137 (126, 149)	143 (132, 154)	**< 0.001**
DBP (mmHg)		81 (75, 88)	80 (72.5, 87)	77 (68, 85)	**< 0.001**
Waist circumstance (cm)		85.20 (8.37)	86.01 (9.18)	88.29 (8.92)	**< 0.001**
TUG test (s)		9.8 (8.9, 10.7)	10.6 (9.4, 12.4)	15.4 (12.3, 19.6)	**< 0.001**
Creatinine (µmol/L)		65 (54, 77)	66 (55, 80)	71 (58, 91)	**< 0.001**
TC (mmol/L)		5.4 (4.6, 6.1)	5.2 (4.4, 6.0)	5.1 (4.2,6.0)	**< 0.001**
TG (mmol/L)		1.5 (1.1, 2.0)	1.4 (1.1, 2.0)	1.5 (1.1, 2.0)	0.90
HDL (mmol/L)		1.2 (1.0, 1.4)	1.2 (1.0, 1.4)	1.2 (1.0, 1.5)	0.33
LDL (mmol/L)		2.8 (2.2, 3.4)	2.7 (2.0, 3.4)	2.7 (2.1, 3.4)	0.18
Fasting blood glucose (mmol/L)		6 (5.5, 6.8)	5.9 (5.5, 6.9)	6.1 (5.3, 7.6)	0.60

ADL: basic activities of daily living, IADL: instrumental activities of daily living, IQR: interquartile range, SSRS: social support rating scale, SBP: systolic blood pressure, DBP: diastolic blood pressure, TUG: timed up and go test, TC: total cholesterol, TG: triglycerides, HDL-C: high-density lipoprotein cholesterol, LDL-C: low-density lipoprotein cholesterol. Malnutrition risk was defined as an MNA-SF score < 12, cognitive impairment as Minicog score ≤ 2, anxiety as GAD-7 > 4, depression as GDS-4 ≥ 2, insomnia as AIS > 3. Comorbidity was defined as the number of the coexistence of chronic conditions

During one year of follow-up, improved, stable, and worsening frailty status was observed in 460 (35.6%), 526 (40.7%), and 306 (23.7%) subjects, respectively. We observed 603 (46.7%) prefrail and 108 (8.35%) frail subjects after one year of follow-up. As shown in Table 2, subjects with a worsening frailty state had a higher proportion of living alone, and hospitalization admission during the one year of follow-up (*P* = 0.046, or *P* < 0.001). They experienced significant ADL/IADL decline and had a higher malnutrition risk (both *P* < 0.001), and had lower DBP and HDL-C levels, and higher levels of fasting blood glucose (all *P* < 0.05).

**Table 2:** Characteristics of subjects by the transition of frailty state

Variables		Stable/improved N = 986	Worsening N = 306	*P* value
**General Characteristics**				
Age (year, ‾x ± s)		72.2 ± 7.2	72.9 ± 7.6	0.17
Female (N, %)		589 (59.7%)	185 (60.5%)	0.84
Hospitalization during one year of follow-up (N, %)		131 (13.3%)	74 (24.2%)	**< 0.001**
Widowed, divorced, or unmarried (N, %)		212 (21.5%)	79 (25.8%)	0.11
Living alone (N, %)		146 (14.8%)	60 (19.6%)	**0.046**
Monthly income < 3000 RMB (N, %)		326 (33.1%)	98 (32.6%)	0.85
Education (primary school or below, N, %)		184 (18.7%)	56 (18.3%)	0.99
Current smoking (N, %)		78 (7.9%)	22 (7.2%)	0.16
Current drinking (N, %)		135 (13.7%)	42 (13.7%)	0.99
**Nutrition & medical condition**				
MNA-SF (median, IQR)		13 (12, 14)	12 (11, 14)	**< 0.001**
Dietary diversity score (median, IQR)		28 (25, 31)	28 (25, 31)	0.99
Hypertension (N, %)		631 (64.0%)	202 (66.0%)	0.52
Diabetes (N, %)		257 (26.1%)	95 (31.0%)	0.087
Coronary heart disease (N, %)		69 (7.0%)	22 (7.2%)	0.91
Stroke (N, %)		39 (4.0%)	20 (6.5%)	0.059
Comorbidity (N, %)		582 (59.0%)	189 (61.8%)	0.39
Polypharmacy (N, %)		293 (29.7%)	98 (32.0%)	0.44
**Mobility & physical function**				
ADL/IADL decline (median, IQR)		0.05 ± 1.79	0.73 ± 2.94	**< 0.001**
Unexplained fall ≥ 2 times in the past one year (N, %)		110 (11.2%)	28 (9.2%)	0.41
Vision impairment (N, %)		60 (6.1%)	14 (4.6%)	0.33
Hearing impairment (N, %)		49 (5.0%)	16 (5.2%)	0.85
Continence (N, %)		93 (9.4%)	36 (11.8%)	0.23
Constipation (N, %)		31 (3.1%)	12 (3.9%)	0.50
SSRS (‾x ± s)		26.9 ± 6.2	26.8 ± 6.7	0.97
**Mental & psychological health**				
Minicog score (median, IQR)		4 (3, 5)	4 (3, 5)	0.81
GAD-7 score (median, IQR)		0 (0, 4)	0 (0, 3)	0.92
GDS-4 score (median, IQR)		0 (0, 1)	0 (0, 1)	0.20
AIS (median, IQR)		2 (0, 6)	2 (0, 6)	0.62
Chronic pain (N, %)		468 (47.5%)	134 (43.8%)	0.27
**Physical Exam**				
SBP (mmHg)		138 (126, 150)	136.5 (127, 148)	0.37
DBP (mmHg)		80.3 ± 10.9	78.7 ± 11.4	**0.038**
BMI (kg/m^2^)		24.6 (22.7, 26.6)	24.6 (22.5, 26.4)	0.89
WC (cm)		86.1 ± 8.8	86.7 ± 9.1	0.34
Heart rate (bpm)		68 (62, 76)	69 (62, 77)	0.54
Time-up and go test (s)		10.3 (9.2, 12.0)	10.3 (8.9, 12.2)	0.72
**Laboratory data**				
Creatinine (µmol/L)		66 (54, 79)	66 (54, 79)	0.87
Uric acid (µmol/L)		366 (309, 428)	364 (318, 418)	0.91
HDL-C (mmol/L)		1.21 (0.99, 1.42)	1.18 (1.10, 1.38)	**0.04**
LDL-C (mmol/L)		2.75 (2.09, 3.43)	2.745 (2.16, 3.4)	0.80
TG (mmol/L)		1.45 (1.07, 2.0)	1.475 (1.09, 2.0)	0.47
TC (mmol/L)		5.26 (4.44, 5.99)	5.32 (4.43, 6.06)	0.50
Fasting blood glucose (mmol/L)		5.9 (5.4, 6.7)	6.1 (5.5, 7.4)	**0.016**
Albumin (g/L)		37.5 ± 8.05	37.5 ± 7.90	1.00
Hemoglobin (g/L)		139 (131, 149)	139 (131, 149)	0.90

ADL: basic activities of daily living, IADL: instrumental activities of daily living, MNA-SF: mini nutritional assessment short form, SSRS: social support rating scale, GAD-7: generalized anxiety disorder-7, GDS-4: geriatric depression scale-4, AIS: Athens insomnia scale, SBP: systolic blood pressure, DBP: diastolic blood pressure, BMI: body mass index, WC: waist circumference, HDL-C: high-density lipoprotein cholesterol, LDL-C: low-density lipoprotein cholesterol, TG: triglycerides, TC: total cholesterol

**Fig. 1:**
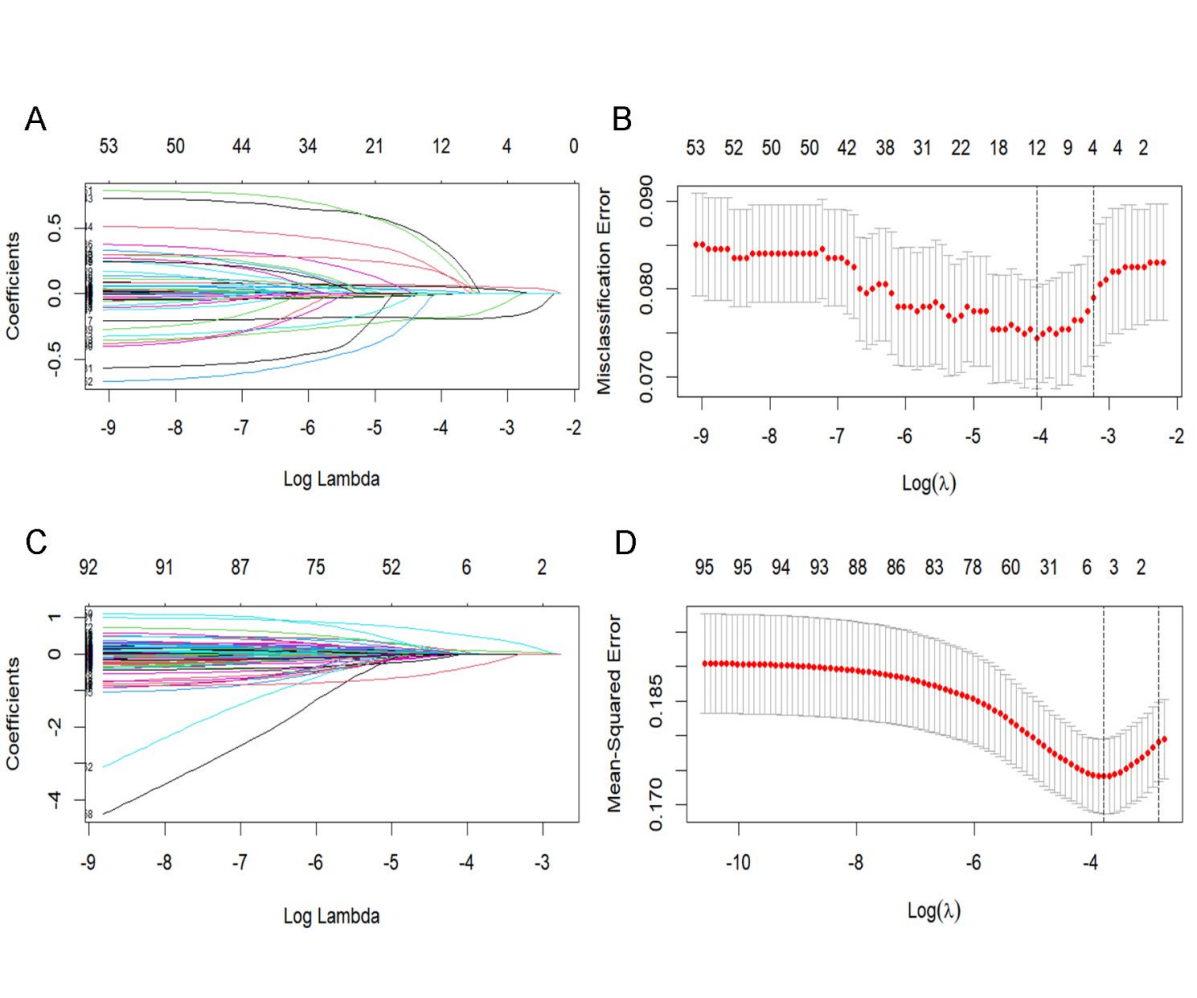
**Results of LASSO regression with 10-fold cross-validation. **A, B: LASSO regression with 10-fold cross-validation for frailty identification; C, D: LASSO regression with 10-fold cross-validation for frailty transition

The results of LASSO regression with 10-fold cross-validation in 1,981 subjects for frailty identification are shown in Fig. 1 (A, B). When λ is taken as the minimum mean square error (MSE) (λ min, 0.0206) and the minimum MSE plus 1 standard error (λ 1se, 0.0328), there were 11 and 4 variables that remained significant associated factors of frailty status, respectively, as shown in Supplementary Table 4. The C-statistics for these two models were compared with the Delong test (Supplementary Table 5). A higher C-statistic was observed in the model with λ min (ΔC-statistics = 0.0189, *P* < 0.001). Calibration plots were built for each model; the model with λ min performed well (all Hosmer-Lemeshow χ^2^ = 14.30, *P* = 0.160, Supplementary Fig. 2A, B). As a result, λ was chosen as λ min. The variables in the prediction of frailty transition were selected in the same way, and the LASSO regression results with 10-fold cross-validation in 1,292 subjects are displayed in Fig. 1 (C, D). When λ min was set, the variables included in the final prediction model were ADL/IADL decline, MNA-SF score, HDL-C, DBP, and hospitalization. The C-statistic was 0.701 for this model, and the calibration was satisfactory (Hosmer-Lemeshow χ^2^ = 11.21, *P* = 0.341, Supplementary Fig. 2C).

**Fig. 2:**
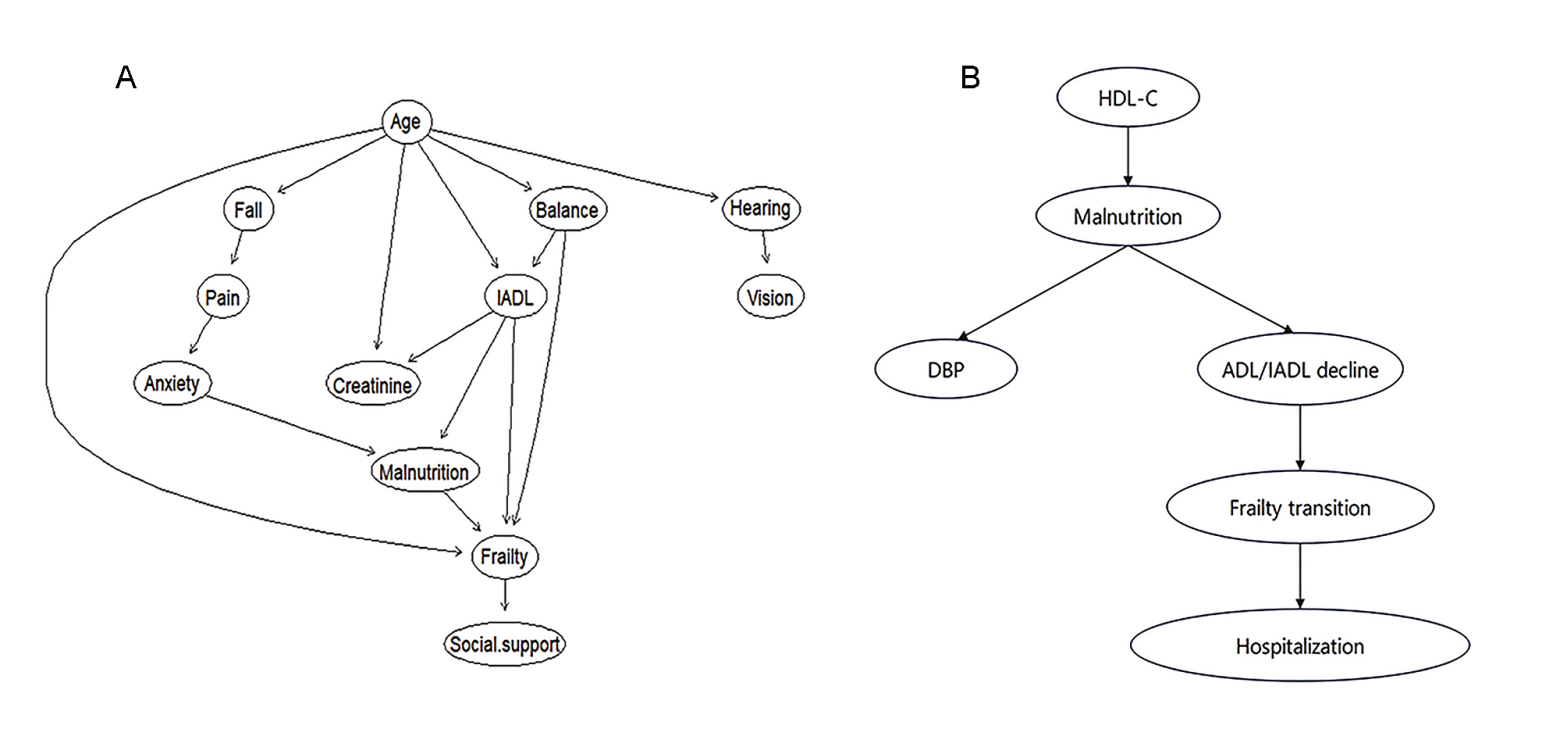
**Bayesian network topology of factors relating to frailty status and frailty transition. **A: Bayesian network topology of factors relating to frailty status; B: Bayesian network topology of predictors relating to frailty transition

After the transformation of categorical variables (Supplementary Table 6), based on the 11 variables screened by LASSO regression, the Bayesian network topology with 12 nodes and 17 directed edges of frailty-related factors was constructed, as shown in Fig. 2A. Figure 2B shows the Bayesian network model presenting the frailty transition and its predictors. Supplementary Tables 7–8 present the conditional probability table (CPT) for frailty identification and frailty transition, which demonstrate the related coefficients among the variables.

**Fig. 3:**
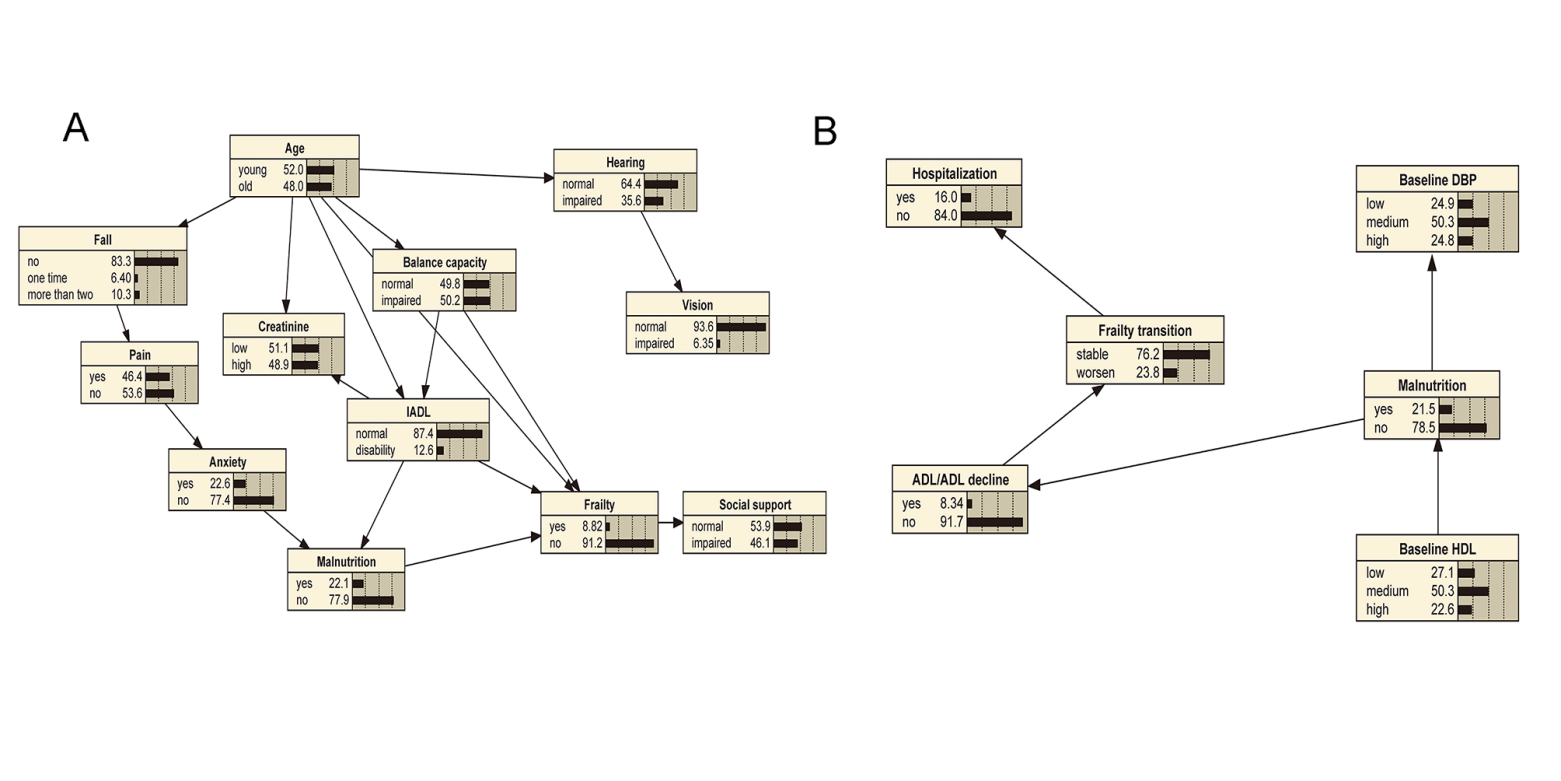
**Risk reasoning of Bayesian network model for frailty identification and frailty transition. **A: Risk reasoning of Bayesian network model for frailty identification; B: Risk reasoning of Bayesian network model for frailty transition. Age: young = ≤ 71 years old, old = > 71 years old; IADL: instrumental activities of daily living, normal: IADL = 8, impaired: IADL < 8; balance capacity: normal: timed up and go test ≤ 10.33s, impaired: timed up and go test > 10.33s; malnutrition: yes: MNA-SF score < 12, no: MNA-SF score ≥ 12; anxiety: yes: GAD-7 > 4, no: GAD-7 ≤ 4; creatine: low: <66µmol/L, high: ≥66µmol/L; social support: normal: social support rating scale ≥ 27, social support rating scale < 27. Hospitalization: hospital admissions within one year; frailty transition: stable/improved or impaired according to the change of the Fried phenotype score; ADL/IADL: basic/instrumental activities of daily living; the change of the ADL/IADL score (14 scores in total) was calculated by baseline scores minus follow-up scores, subjects who received positive scores were considered as “ADL/IADL decline”; malnutrition: yes: MNA-SF score < 12, no: MNA-SF score ≥ 12; baseline DBP: low: <73 mm Hg, medium: 73 mm Hg ≤ DBP ≤ 87 mm Hg, high: > 87 mm Hg; baseline HDL: low: <0.99 mmol/L, medium: 0.99 mmol/L ≤ HDL ≤ 1.42 mmol/L, high: >1.42 mmol/L

As shown in Fig. 3, the Bayesian network model can be used to quantitatively analyze the impact of associated factors on frailty status or frailty transition by computing conditional probabilities. For instance, if an individual is older than 71 years, the probability of developing frailty is 14.4%; if the individual also has balance impairment, the probability increases to 20.2%. The likelihood of frailty reaches 53.2% if this person also has IADL disability and malnutrition. Similarly, if a person is without the above-mentioned risk factors, the probability of frailty is 0.56% (Supplementary Fig. 3). Bayesian network analysis can also be used to understand the inter-relationship between associated factors. For example, as shown in Supplementary Fig. 4, if a person has malnutrition, the probability of ADL/IADL decline within one year is increased to 12.2% compared to 7.27% without malnutrition. In addition, if a person has low levels of HDL-C, the probability of malnutrition increases to 31% compared to 16.3% with high levels of HDL-C (Supplementary Fig. 5).

## Discussion

Based on Bayesian network analysis, this study found that age, nutritional status, IADL, balance capacity, and social support were directly related to frailty status in a community-dwelling older population. ADL/IADL decline was a direct predictor of a worsening in frailty, which further increased the risk of hospitalization within one year. We first investigated the factors related to the identification and prediction of frailty at a cross-sectional and longitudinal level, which may provide useful information in risk stratification and early intervention regarding frailty in older community-dwelling residents.

We found that 23.7% of the subjects experienced a deterioration in the frailty state over a period of one year. The rate of deterioration in the frailty state was comparable to other studies with similar follow-up time [22, 23].

Our findings suggest that age was a major contributor to frailty at a cross-sectional level. It not only directly affected the occurrence of frailty, but also exerted an influence on balance capacity, IADL, the risk of falling, and was ultimately related to frailty. Almost all of previous literature has claimed a correlation between aging and frailty status [24]. Some studies have suggested that the relationship between frailty and age is not simply linear. In fact, frailty increases exponentially with an increase in age [25], which is mainly attributed to the degenerative alterations of body organs and the subsequent decline of physical reserve capacity during the process of aging. At a longitudinal level, we did not find that age was not linked with the frailty transition, as measured by the Fried phenotype. Other studies, for example Pegorari et al., observed that older age was predictive of the pre-frail and frail state [26]. This inconsistent result between the cross-sectional and longitudinal levels were not unexpected, given that one-year of follow-up was relatively short, and the impacts of aging on frailty needed to be assessed over a longer period of time.

We also discovered that there was an interaction between balance capacity and frailty. Normal aging is accompanied by a loss of hair cells and afferent fibers [27], as well as a deterioration in the neuromuscular, somatosensory, and cognitive systems [28]. Lacroix et al. reported that, compared to unsupervised programs, supervised balance programs significantly improve measures of balance and muscle strength in the older adults [29].

The results of the Bayesian network analysis indicated that frailty had a direct impact on social support, which is consistent with previous studies [7, 30]. This phenomenon may be partially owing to the decrease in social participation and loss of social belonging caused by frailty. The decline of physical and cognitive functions in frail older people can easily result in social withdrawal, which not only affects objective and subjective support, but also reduces the actual use of social support in older adults. Additionally, frail older people become more dependent on their families, but family members may be prone to criticism and opposition to the older people due to the long-term care burden, which also leads to an increase of family discord and contradiction [31].

Regarding the factors predicting frailty transition, we observed that IADL was associated with frailty status, and ADL/IADL decline within one year predicted the worsening of frailty. These results are in line with existing knowledge that ADL and IADL are the most important influencing factors of frailty, implying that physical function plays an important role in the occurrence and development of frailty [32, 33]. Physical function reflects the status of lower limb muscle strength and systemic muscle function, which is closely related to sarcopenia, as an integral correlate of the physical component of frailty [34]. Furthermore, a decline in physical function is closely related to comorbidities, polypharmacy, and cognitive impairment, which then exacerbate the progression of frailty [35].

It has been found that worsening frailty could lead to an increased risk of hospitalization. A meta-analysis that encompassed 11 articles suggested that a frail status exhibited a 1.23-fold increased risk for hospitalization [36]. Interestingly, Landré et al. reported that multiple hospitalization episodes, and hospitalization in emergency were significantly associated with transitions from robust health towards frailty [37]. The above-mentioned evidence illustrates that a change in frailty and hospitalization may involve a cause-and-effect relationship on both sides.

As shown in our study, malnutrition as reflected in the low MNA-SF score was discovered to be a direct risk factor of frailty status, and indirectly related to the deterioration in frailty state through an ADL/IADL decline. The close association between nutrition and frailty has also been confirmed by many studies [34, 38]. Malnutrition worsens frailty by damaging the immune system and triggering infections, which may result in multi-system dysfunction. Decreased muscle protein synthesis due to insufficient protein or energy intake in malnourished individuals can lead to decreased muscle strength and sarcopenia [39]. Nutritional interventions with and without physical activity programs can prevent or even reverse the progression of frailty and sarcopenia [34, 40].

We also discovered that some of the variables were inter-related, such as nutritional status and activities of daily living. According to the Bayesian network structure for frailty identification and frailty transition, malnutrition and ADL/IADL were each other’s parent or child node. Liu et al. observed that being dependent in ADL and a lower score of ADL were independent factors of malnutrition in older Chinese inpatients [41], which possibly related to the fact that impaired functional capacity usually causes a loss of appetite and reduced food intake. On the other hand, it has been reported that, in geriatric rehabilitation inpatients, malnutrition at admission was associated with “remained poor” and “deteriorated” ADL trajectories from two weeks pre-admission to three months post-discharge [42], which implied that decreased muscle mass caused by malnutrition could result in reduced physical function. Therefore, we believe that the relationship between decreased physical capacity and malnutrition is bidirectional.

A relationship between malnutrition and DBP levels has been discovered. Nutritional status plays a key role in blood pressure regulation. Lower DBP levels may be indicative of reduced organ perfusion and orthostatic hypotension, which are known associated factors for malnutrition or risk of malnutrition [43, 44]. Evidence of the association with HDL-C levels and nutritional status is sparse. It was reported by Formiga et al. that, in 85-year-old subjects, lower HDL-C values were associated with poor MNA scores in the bivariate analysis, but not in the multivariate analysis. Higher levels of HDL-C were associated with better ADL performance evaluated by the Barthel index after adjustment for covariates [45]. We speculate that low HDL-C levels increase the risk of ischemic peripheral disease, leading to a decline in lower extremity performance and impaired functional capacity, which indirectly relates to malnutrition. However, the underlying mechanism for the association between HDL-C levels and malnutrition need to be further clarified.

In addition to the above direct influencing factors of frailty, anxiety, vision and hearing, risk of falling, chronic pain, and serum creatinine levels were indirectly associated with frailty, in accordance with other studies [46–49]. Although many influencing factors of frailty have been explored, the underlying pathophysiological mechanism of frailty is still unclear. Biological mechanisms of the aging process may be involved [50].

Our study supplements the existing knowledge of the associated factors in the identification of frailty status and the prediction of frailty transition. The use of Bayesian network analysis allowed the associated variables and outcomes to be modeled simultaneously and the interactions between variables to be studied thoroughly [51]. However, there were some limitations that are worth mentioning. Firstly, those subjects who were excluded for not completing two visits tended to be more solitary than those who completed the visits, but they were comparable in other clinical aspects. Secondly, we established a discrete Bayesian network that involved discretization for the continuous variables, which may result in a loss of information. However, discretization is a common practice in risk prediction, probably due to its higher clinical applicability. Thirdly, we collected data from one community center located in Fuzhou City, so extrapolations from our findings to other population require further evaluation. Lastly, the accuracy and applicability of the Bayesian network structure still need to be continuously adjusted and verified in external cohorts with larger sample sizes.

Several future research implications can be generated. Identifying the influencing factors of frailty is crucial for the timely development of frailty prevention and intervention measures. Among the direct influencing factors of frailty, i.e. age, IADL and ADL, nutritional status, and balance capacity, all of them are modifiable factors except for age, and thus may can become potential targets for frailty intervention measures. Maintaining an intact physical-nutrition reserve and functioning throughout the process of ageing prevents the progression of frailty [22].

## Conclusion

Age, nutritional status, balance capacity, IADL, and social support are associated with frailty state. The identified predictors for worsening frailty status in one year is an ADL / IADL decline, which further leads to an increased risk of hospitalization. Early identification of the frailty and proper prediction of the frailty transition both facilitate effective and precise intervention, and contribute to the improvement of health status in the older population.

## Electronic supplementary material

Below is the link to the electronic supplementary material.

Supplementary Material 1. Supplementary Tables 1-8; Supplementary Figures 1-5.

## Data Availability

The datasets analysed during the current study are not publicly available. We cannot publish public data because Fujian Provincial Hospital and Shengli Clinical Medical College of Fujian Medical University, which own the raw data, do not agree with publishing the data. But the data are available upon reasonable request and with permission of the corresponding authors.

## Declarations

This study was evaluated and approved by the Ethics Committee of Fujian Provincial Hospital (No. K2020-05-008) and was conducted according to the principles of the Declaration of Helsinki. Written informed consent was obtained from all participants prior to their enrollment.

## Abbreviations

ADL: basic activities of daily living
IADL: instrumental activities of daily living
MNA-SF: mini nutritional assessment short form
SSRS: social support rating scale
GAD-7: generalized anxiety disorder-7
GDS-4: geriatric depression scale-4
AIS: Athens insomnia scale
LASSO: Least absolute shrinkage and selection operator.
