# Supplementary material for: The identification and prediction of frailty based on Bayesian network analysis in a community-dwelling older population
Source: BMC Geriatr. 2022 Nov 11;22:847. doi: 10.1186/s12877-022-03520-7 (PMC9652858; doi:10.1186/s12877-022-03520-7)
Supplement: Supplementary file 1 — Supplementary Material 1. Supplementary Tables 1-8; Supplementary Figures 1-5. [file 12877_2022_3520_MOESM1_ESM.docx]

**The identification and prediction of frailty based on Bayesian network analysis in a community-dwelling older population**

Yin Yuan, Siyang Lin, Xiaoming Huang, Na Li, Jiaxin Zheng, Feng Huang, and Pengli Zhu

Additional file 1 contains eight supplementary tables and five supplementary figures.

**Contents:**

**Supplementary Table 1** Characteristics of subjects who did or did not complete two visits

**Supplementary Table 2** Characteristics of subjects at baseline and after one year

**Supplementary Table 3** Variables included in the LASSO regression

**Supplementary Table 4** LASSO regression with 10-fold cross-validation in 1,981 subjects for frailty identification

**Supplementary Table 5** Comparison of C-statistics of LASSO regression

**Supplementary Table 6** Categorical variable transformation for Bayesian network

**Supplementary Table 7** Condition Probability Table for frailty identification

**Supplementary Table 8** Condition Probability Table for frailty transition

**Supplementary Figure 1** Flowchart of the study

**Supplementary Figure 2** Calibration plots of LASSO regression

**Supplementary Figure 3** Risk reasoning of Bayesian network for frailty identification

**Supplementary Figure 4** Bayesian network I under known evidence variable for frailty transition

**Supplementary Figure 5** Bayesian network II under known evidence variable for frailty transition

**Supplementary Table 1 Characteristics of subjects who did or did not complete two visits**

| **Variables** | | **Did not complete**  **(N = 689)** | **Completed**  **(N = 1,292)** | ***P* value** |
| --- | --- | --- | --- | --- |
| **Demographics** | |  |  |  |
| Female (n, %) | | 412 (59.8%) | 777 (60.1%) | 0.32 |
| Age (year, ‾x±s) | | 72.47 ± 7.28 | 72.50 ± 7.06 | 0.92 |
| Living alone (N, %) |  | 76 (11.0%) | 184 (14.2%) | **0.024** |
| Widowed/divorced/unmarried (N, %) | | 148 (21.5%) | 275 (21.3%) | 0.81 |
| Former/current smoking (N, %) | | 122 (17.7%) | 190 (14.7%) | 0.15 |
| Former/current drinking (N, %) | | 108 (15.7%) | 174 (13.5%) | 0.29 |
| **Clinical factors** | |  |  |  |
| Hypertension (N, %) | | 424 (61.5%) | 818 (63.3%) | 0.14 |
| Diabetes (N, %) | | 216 (31.3%) | 421 (32.6%) | 0.31 |
| Comorbidity (median, IQR) | | 2 (1, 3) | 2 (1, 3) | 0.067 |
| Polypharmacy (N, %) | | 198 (28.7%) | 381 (29.5%) | 0.44 |
| **Physical function** | |  |  |  |
| ADL (median, IQR) | | 6 (6, 6) | 6 (6, 6) | 0.11 |
| IADL (median, IQR) | | 8 (8, 8) | 8 (8, 8) | 0.072 |
| Robust (N, %) | | 236 (34.2%) | 482 (37.3%) | 0.071 |
| Prefrailty (N, %) | | 386 (56.1%) | 669 (51.8%) |  |
| Frailty (N, %) | | 67 (9.7%) | 141 (10.9%) |  |

ADL: basic activities of daily living, IADL: instrumental activities of daily living.

**Supplementary Table 2 Characteristics of subjects at baseline and after one year**

| **Variables** | | **Baseline**  **(N = 1,981)** | **One year of follow-up (N = 1,292)** |
| --- | --- | --- | --- |
| Female (n, %) | | 1189 (60.0%) | 774 (59.9%) |
| Age (year, ‾x±s) | | 72.49 ± 7.14 | 72.37 ± 7.27 |
| Living status (N, %) | Living with families | 1721 (86.9%) | 1085 (84.0%) |
|  | Living with others | 133 (6.7%) | 120 (9.3%) |
|  | Living alone | 127 (6.4%) | 87 (6.7%) |
| Marital status (N, %) | Married | 1558 (78.6%) | 1001 (77.5%) |
|  | Widowed/divorced/unmarried | 423 (21.4%) | 291 (22.5%) |
| Monthly income (N, %) | <3000 RMB | 687 (34.9%) | 424 (32.8%) |
|  | 3000 RMB ~ | 1087 (55.2%) | 739 (57.3%) |
|  | >6000 RMB | 194 (9.9%) | 129 (9.9%) |
| Comorbidity (median, IQR) | | 2 (1, 3) | 2 (1, 3) |
| Polypharmacy (N, %) | | 579 (29.2%) | 391 (30.3%) |
| Fried phenotye components (N, %) | 0 | 776 (39.2%) | 581 (45.0%) |
|  | 1 | 709 (35.8%) | 404 (31.3%) |
|  | 2 | 331 (16.7%) | 199 (15.4%) |
|  | 3 | 124 (6.26%) | 66 (5.11%) |
|  | 4 | 40 (2.02%) | 35 (2.71%) |
|  | 5 | 1 (0.00%) | 7 (0.5%) |

IQR: interquartile range

**Supplementary Table 3 Variables included in the LASSO regression**

| **Variables** | **Scoring** |
| --- | --- |
| **Categorical variable** |  |
| Gender | Male=1, female=2 |
| Marital status | Unmarried/widowed/divorced=1, married=0 |
| Living alone | Yes=1, no=0 |
| Manual labor or unemployed | Yes=1, no=0 |
| Education | Primary school or below=2, junior/senior high school=1, bachelor degree or above=0 |
| Smoking | Former/current smoking=1, never=0 |
| Drinking | Former/current drinking=1, never=0 |
| Grain intake | Daily consumption = 4, weekly consumption = 3, monthly consumption = 2, yearly consumption = 1, not eaten =0 |
| Vegetable intake | Daily consumption = 4, weekly consumption = 3, monthly consumption = 2, yearly consumption = 1, not eaten =0 |
| Fruit intake | Daily consumption = 4, weekly consumption = 3, monthly consumption = 2, yearly consumption = 1, not eaten =0 |
| Soy products intake | Daily consumption = 4, weekly consumption = 3, monthly consumption = 2, yearly consumption = 1, not eaten =0 |
| Poultry/meat intake | Daily consumption = 4, weekly consumption = 3, monthly consumption = 2, yearly consumption = 1, not eaten =0 |
| Seafood intake | Daily consumption = 4, weekly consumption = 3, monthly consumption = 2, yearly consumption = 1, not eaten =0 |
| Dairy intake | Daily consumption = 4, weekly consumption = 3, monthly consumption = 2, yearly consumption = 1, not eaten =0 |
| Egg intake | Daily consumption = 4, weekly consumption = 3, monthly consumption = 2, yearly consumption = 1, not eaten =0 |
| Hypertension | Yes=1, no=0 |
| Diabetes | Yes=1, no=0 |
| Coronary heart disease | Yes=1, no=0 |
| **Variables** | **Scoring** |
| Stroke | Yes=1, no=0 |
| Cancer | Yes=1, no=0 |
| Respiratory system disease | Yes=1, no=0 |
| Chronic kidney disease | Yes=1, no=0 |
| Osteoporosis | Yes=1, no=0 |
| Digestive system disease | Yes=1, no=0 |
| Antihypertensive agent | Yes=1, no=0 |
| Antiplatelet agent | Yes=1, no=0 |
| Hypoglycemic drugs | Yes=1, no=0 |
| Lipid-lowering medicine | Yes=1, no=0 |
| Sedatives | Yes=1, no=0 |
| Vision | Severe impaired = 2, mild impaired = 1, normal= 0 |
| Hearing | Severe impaired = 2, mild impaired = 1, normal= 0 |
| Oral conditions | Severe impaired = 2, mild impaired = 1, normal= 0 |
| Choking | Yes=1, no=0 |
| Constipation | Yes=1, no=0 |
| Continence | Yes=1, no=0 |
| Unexplainable fall in the past year | Yes=1, no=0 |
| Household elevator | Yes=1, no=0 |
| Residential greenspace | Yes=1, no=0 |
| Hospital admission (follow-up data) | Yes=1, no=0 |
| **Continuous variable** |  |
| Age | Year |
| Body mass index | Kg/m^2^ |
| Systolic blood pressure (SBP) | MmHg |
| Diastolic blood pressure (DBP) | MmHg |
| Heart rate | Bpm |
| Timed up and go test | Second |
| Comorbidity | Number |
| Polypharmacy | Number |
| Minicog score | Number |
| Diversity diet score | Number |
| MNA-SF score | Number |
| GAD-7 score | Number |
| GDS-4 score | Number |
| AIS | Number |
| Pain rating | Number |
| **Continuous variable** | **Scoring** |
| SSRS | Number |
| ADL | Number |
| IADL | Number |
| ADL/IADL decline (follow-up data) | Number |
| Hemoglobin | G/L |
| Albumin | G/L |
| Creatinine | μmol/L |
| Uric acid | μmol/L |
| Total cholesterol (TC) | Mmol/L |
| Triacylglycerol (TG) | Mmol/L |
| Low density lipoprotein cholesterol (LDL-C) | Mmol/L |
| High density lipoprotein cholesterol (HDL-C) | Mmol/L |
| Fasting glucose | Mmol/L |

MNA-SF: mini nutritional assessment, short form, GAD-7: generalized anxiety disorder-7, GDS-4: geriatric depression scale-4, AIS: Athens insomnia scale, SSRS: social support rating scale, ADL: basic activities of daily living, IADL: instrumental activities of daily living.

**Supplementary Table 4 LASSO regression with 10-fold cross-validation in 1,981 subjects for frailty identification**

| **Model** | **λ** | **No. of variables** | **Variables** |
| --- | --- | --- | --- |
| λ min | 0.0206 | 11 | Age, MNA-SF score, IADL, TUG, GAD-7, SSRS, creatine, vision, hearing, fall, chronic pain |
| λ 1se | 0.0328 | 4 | Age, MNA-SF score, IADL, TUG |

MNA-SF: mini nutritional assessment short form, IADL: instrumental activities of daily living, TUG: timed up and go test, GAD-7: generalized anxiety disorder-7, SSRS: social support rating scale.

**Supplementary Table 5 Comparison of C-statistics of LASSO regression**

| **Models** | **C-statistics** | **SE** | | **95% CI** |
| --- | --- | --- | --- | --- |
| λ min | 0.898 | 0.0116 | | 0.883 - 0.911 |
| λ 1se | 0.879 | 0.0130 | | 0.864 - 0.893 |
| Comparison with Delong test | | | | |
| ΔC-statistics | | | 0.0189 | |
| SE | | | 0.00456 | |
| 95%CI | | | 0.00999 - 0.0279 | |
| *P* value | | | < 0.0001 | |

SE: standard error, CI: confidence interval.

**Supplementary Table 6 Categorical variable transformation for Bayesian network**

| **Variables** | **Scoring** |
| --- | --- |
| Frailty | Yes, no |
| Age | ≤ 71 years old, >71 years old |
| Malnutrition | Yes (MNA-SF <12), no (MNA-SF ≥12) |
| IADL | Normal (IADL = 8), impaired (IADL < 8) |
| Balance | Normal (TUG ≤10.33s), impaired (TUG >10.33s) |
| Anxiety | Yes (GAD-7 >4), no (GAD-7 ≤4) |
| Social support | Normal (SSRS ≥27), impaired (SSRS <27) |
| Creatine | Low (Creatine <66µmol/L), high (Creatine ≥66µmol/L) |
| Vision | Normal, impaired |
| Hearing | Normal, impaired |
| Unexplained falls in the past one year | No, one time, more than one time |
| Chronic pain | Yes, no |
| Hospital admission during one year of follow-up | Yes, no |
| HDL-C levels | Low (< 0.99mmol/L), medium (0.99 mmol/L≤ HDL-C ≤1.42 mmol/L), high (> 1.42mmol/L) |
| DBP levels | Low (<73 mm Hg), medium (73 mmHg ≤ DBP ≤ 87mmHg), high (> 87mmHg) |
| ADL/IADL decline | Yes, no |
| Frailty transition | Worsening, stable/improved |

MNA-SF: mini nutritional assessment short form, ADL: basic activities of daily living, IADL: instrumental activities of daily living, TUG: timed up and go test, GAD-7: generalized anxiety disorder-7, SSRS: social support rating scale, HDL-C: high-density lipoprotein cholesterol, DBP: diastolic blood pressure.

**Supplementary Table 7 Condition Probability Table for frailty identification**

| **Age** | **IADL** | **Balance** | **Malnutrition** | **Frailty** | |
| --- | --- | --- | --- | --- | --- |
|  |  |  |  | **Yes** | **No** |
| ≤71 years old | Normal | Normal | Yes | 0.06 | 0.94 |
| ≤71 years old | Normal | Normal | No | 0.006 | 0.994 |
| ≤71 years old | Normal | Impaired | Yes | 0.16 | 0.84 |
| ≤71 years old | Normal | Impaired | No | 0.04 | 0.96 |
| ≤71 years old | Impaired | Normal | Yes | 0.14 | 0.86 |
| ≤71 years old | Impaired | Normal | No | 0.03 | 0.97 |
| ≤71 years old | Impaired | Impaired | Yes | 0.27 | 0.73 |
| ≤71 years old | Impaired | Impaired | No | 0.11 | 0.89 |
| >71 years old | Normal | Normal | Yes | 0.05 | 0.95 |
| >71 years old | Normal | Normal | No | 0.02 | 0.98 |
| >71 years old | Normal | Impaired | Yes | 0.20 | 0.80 |
| >71 years old | Normal | Impaired | No | 0.11 | 0.89 |
| >71 years old | Impaired | Normal | Yes | 0.11 | 0.89 |
| >71 years old | Impaired | Normal | No | 0.07 | 0.93 |
| >71 years old | Impaired | Impaired | Yes | 0.53 | 0.47 |
| >71 years old | Impaired | Impaired | No | 0.32 | 0.68 |

IADL: instrumental activities of daily living

**Supplementary Table 8 Condition Probability Table for frailty transition**

| **ADL/IADL decline** | **Hospitalization** | **Frailty transition** | |
| --- | --- | --- | --- |
|  |  | **Stable/improved** | **Worsen** |
| Yes | Yes | 0.398 | 0.602 |
| Yes | No | 0.580 | 0.420 |
| No | Yes | 0.663 | 0.337 |
| No | No | 0.804 | 0.196 |

ADL: basic activities of daily living, IADL: instrumental activities of daily living

**
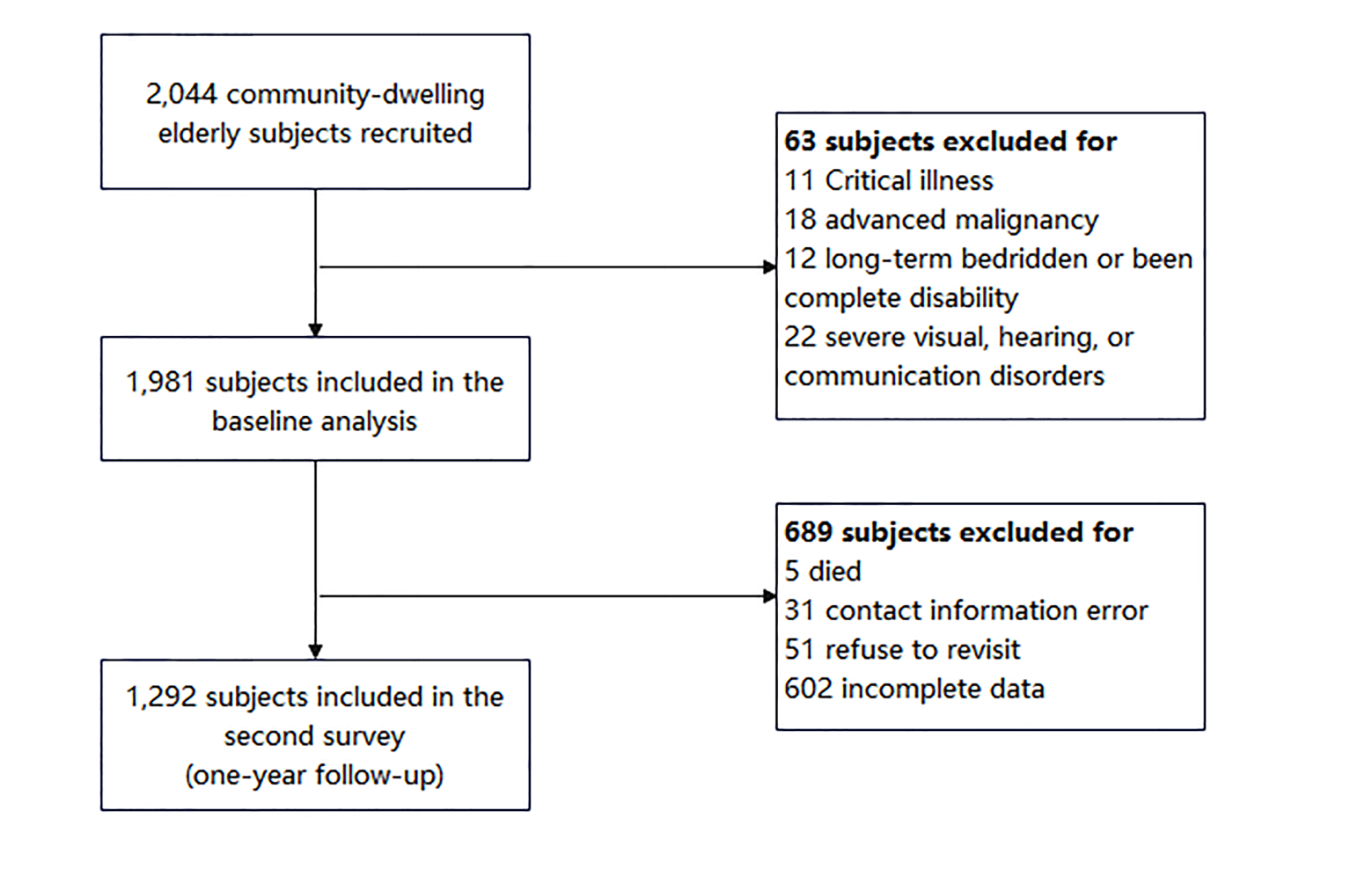
**

**Supplementary Figure 1** Flowchart of the study


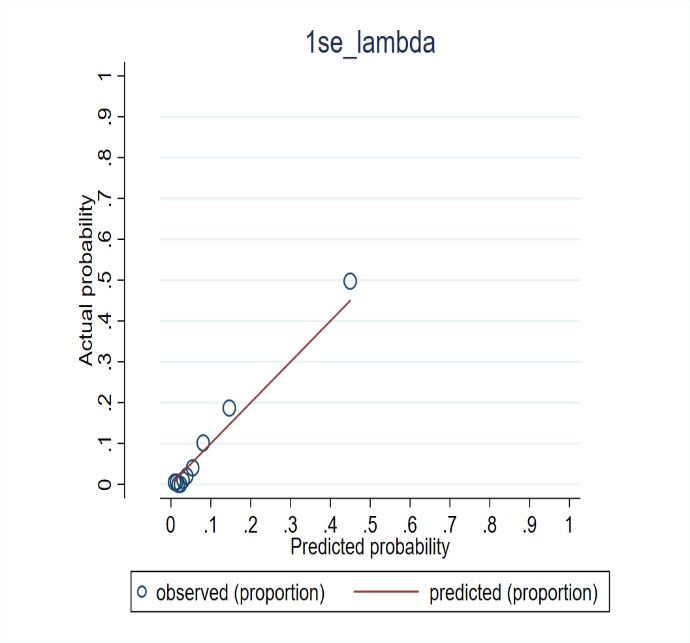

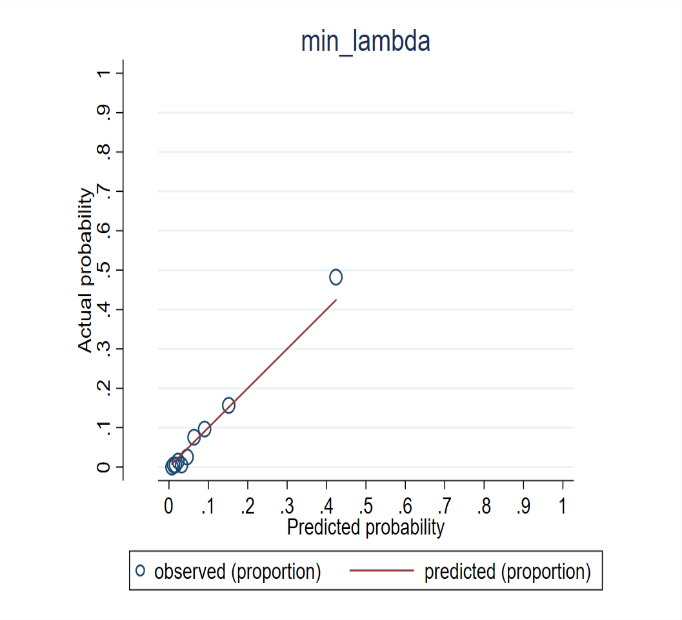

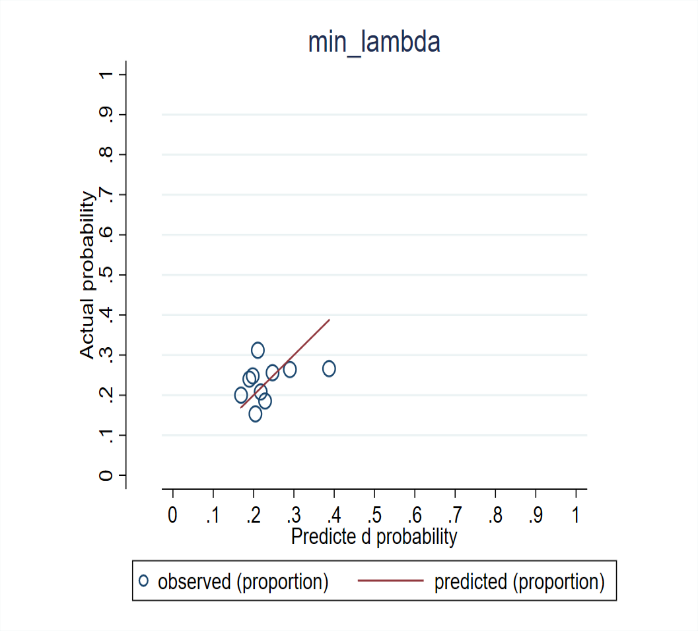


A

B

C

χ^2^= 14.30, *P*=0.160 χ^2^= 20.99, *P*=0.021 χ^2^= 11.21, *P*= 0.341

**Supplementary Figure 2 Calibration plots of LASSO regression**

A, B: LASSO regression for frailty identification, C: LASSO regression for frailty transition.


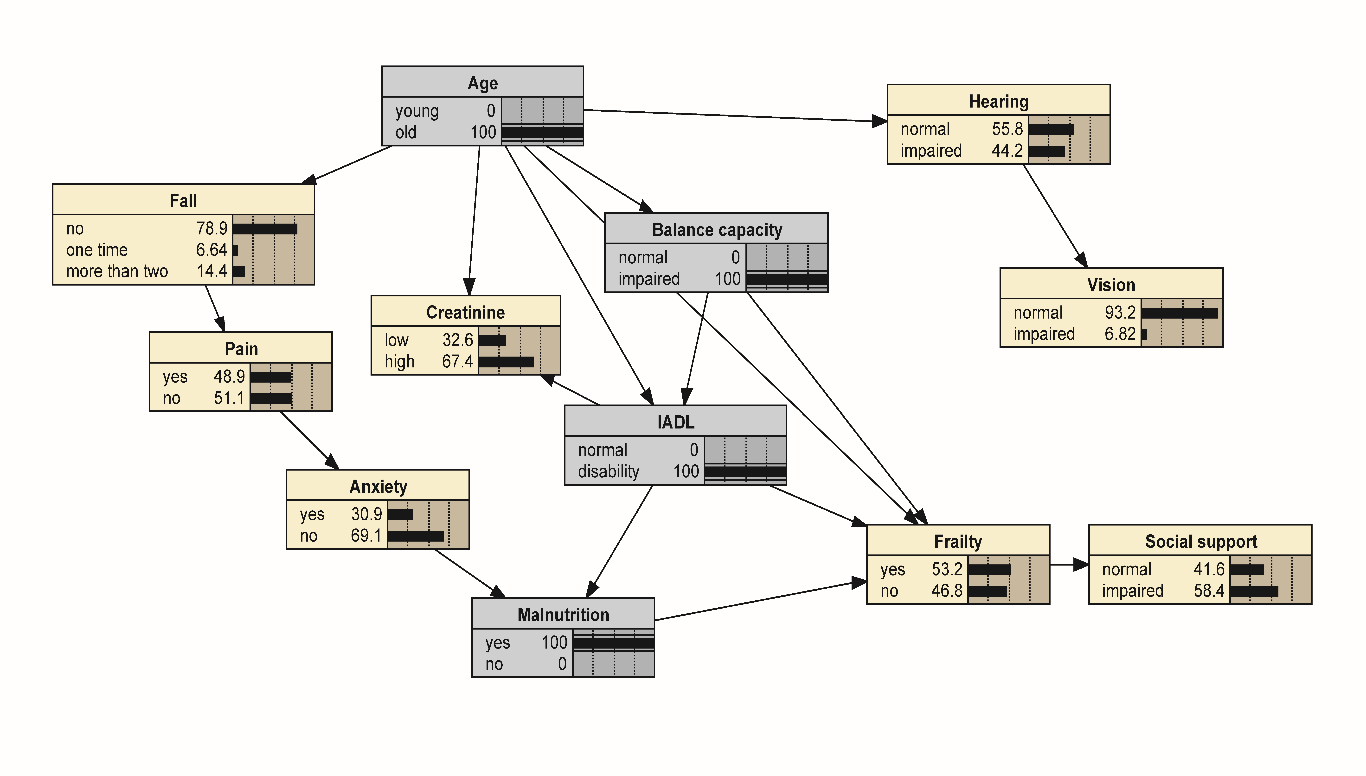


A

**
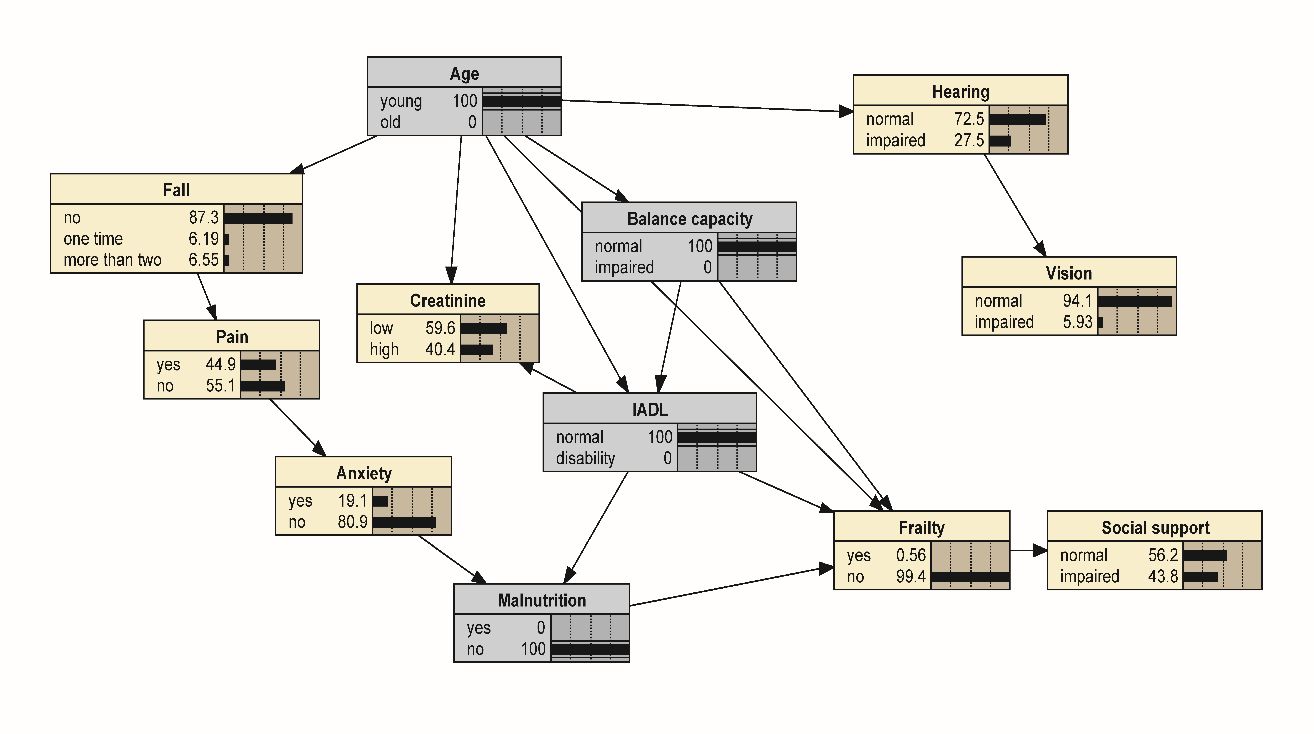
**

B

**Supplementary Figure 3 Risk reasoning of Bayesian network for frailty identification**


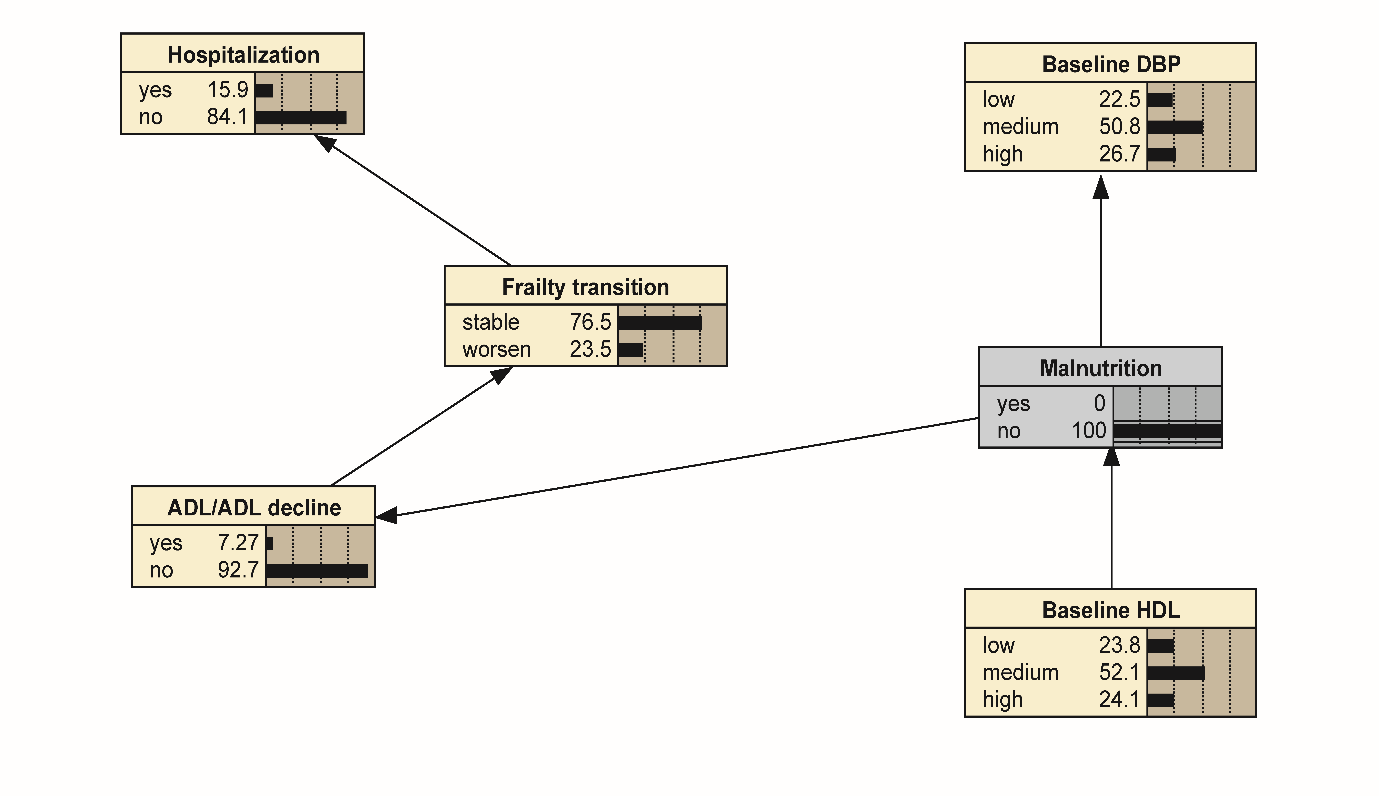


A


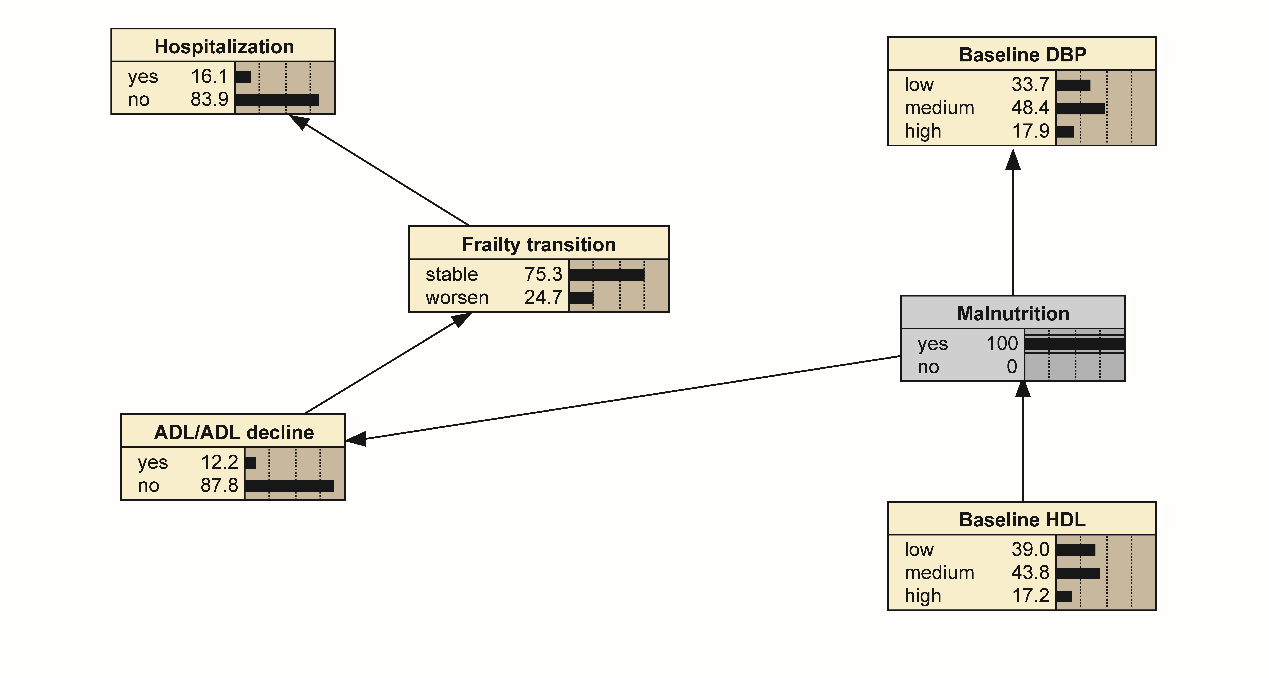


B

**Supplementary Figure 4 Bayesian network I under known evidence variable for frailty transition**


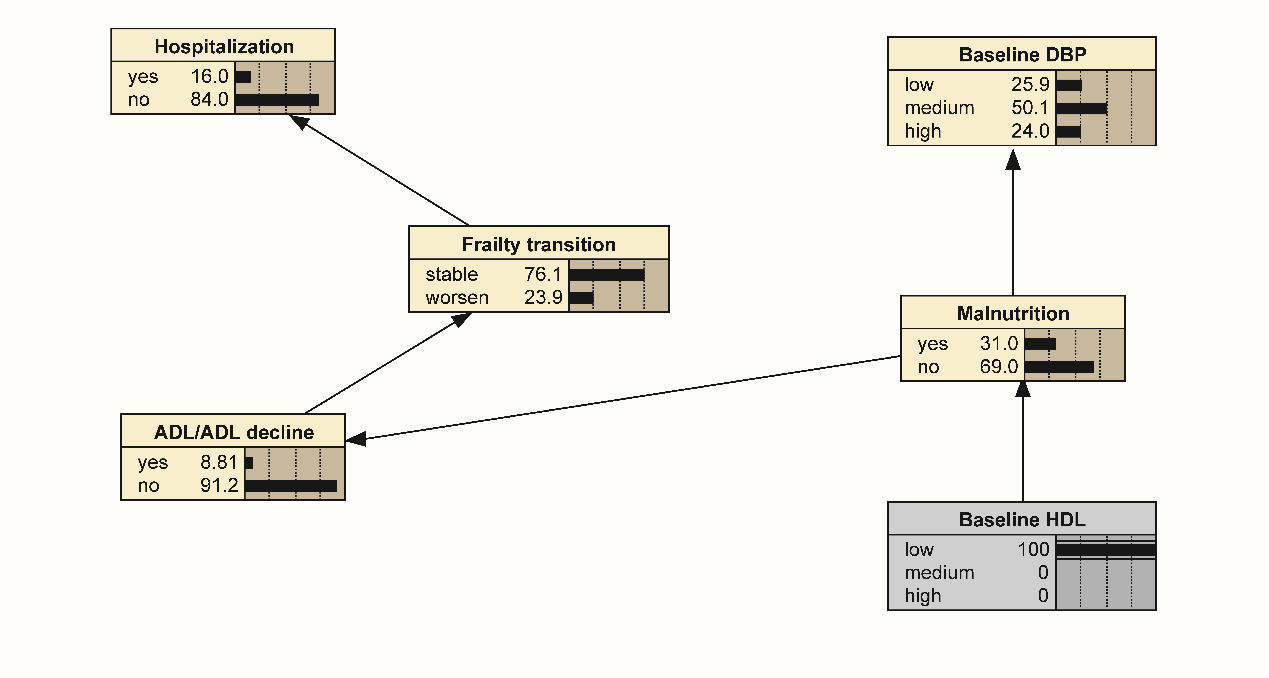


A


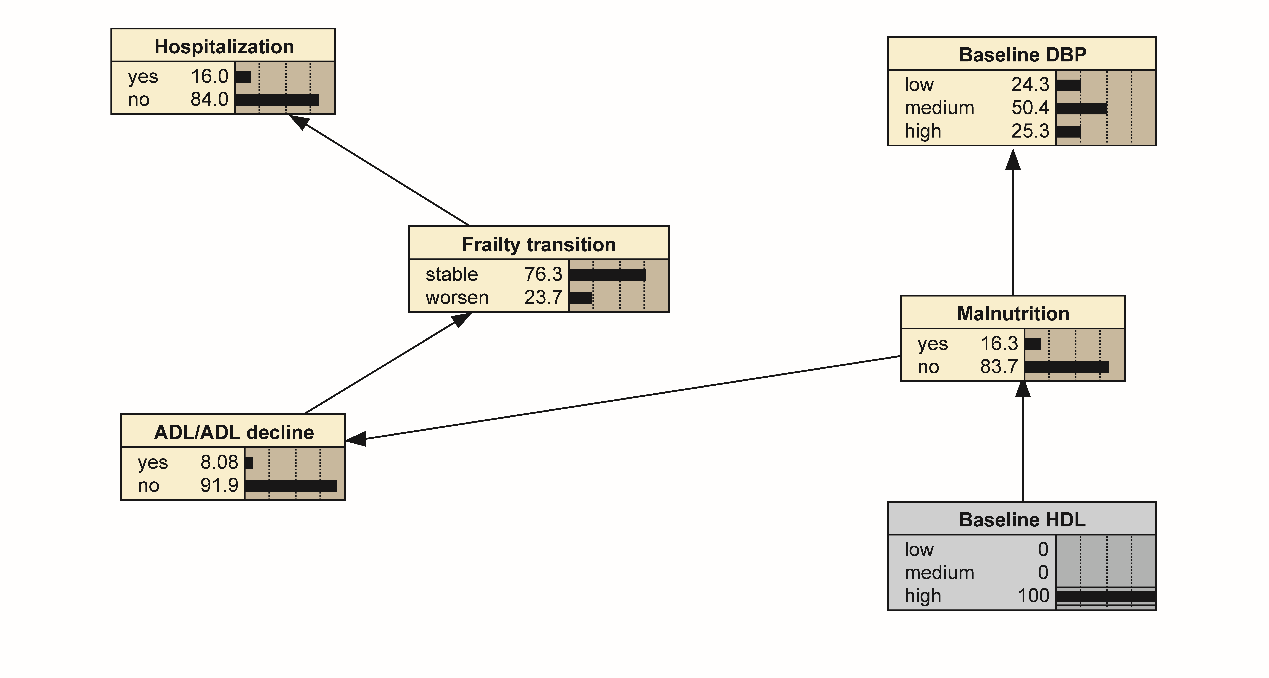


B

**Supplementary Figure 5 Bayesian network II under known evidence variable for frailty transition**
